# T-CAST: An optimized CAST-Seq pipeline for TALEN confirms superior safety and efficacy of obligate-heterodimeric scaffolds

**DOI:** 10.3389/fgeed.2023.1130736

**Published:** 2023-02-20

**Authors:** Manuel Rhiel, Kerstin Geiger, Geoffroy Andrieux, Julia Rositzka, Melanie Boerries, Toni Cathomen, Tatjana I. Cornu

**Affiliations:** ^1^ Institute for Transfusion Medicine and Gene Therapy, Medical Center-University of Freiburg, Freiburg, Germany; ^2^ Center for Chronic Immunodeficiency (CCI), Medical Center-University of Freiburg, Freiburg, Germany; ^3^ Ph.D. Program, Faculty of Biology, University of Freiburg, Freiburg, Germany; ^4^ Institute of Medical Bioinformatics and Systems Medicine, Medical Center-University of Freiburg, Faculty of Medicine, University of Freiburg, Freiburg, Germany; ^5^ German Cancer Consortium (DKTK), Partner Site Freiburg, and German Cancer Research Center (DKFZ), Heidelberg, Germany; ^6^ Faculty of Medicine, University of Freiburg, Freiburg, Germany; ^7^ Comprehensive Cancer Center Freiburg (CCCF), Medical Center—University of Freiburg, Freiburg, Germany

**Keywords:** TALEN, designer nuclease, chromosomal translocations, off-target effects, FokI, preclinical risk assessment, chromosomal rearrangements, obligate heterodimer

## Abstract

Transcription activator-like effector nucleases (TALENs) are programmable nucleases that have entered the clinical stage. Each subunit of the dimer consists of a DNA-binding domain composed of an array of TALE repeats fused to the catalytically active portion of the FokI endonuclease. Upon DNA-binding of both TALEN arms in close proximity, the FokI domains dimerize and induce a staggered-end DNA double strand break. In this present study, we describe the implementation and validation of TALEN-specific CAST-Seq (T-CAST), a pipeline based on CAST-Seq that identifies TALEN-mediated off-target effects, nominates off-target sites with high fidelity, and predicts the TALEN pairing conformation leading to off-target cleavage. We validated T-CAST by assessing off-target effects of two promiscuous TALENs designed to target the *CCR5* and *TRAC* loci. Expression of these TALENs caused high levels of translocations between the target sites and various off-target sites in primary T cells. Introduction of amino acid substitutions to the FokI domains, which render TALENs obligate-heterodimeric (OH-TALEN), mitigated the aforementioned off-target effects without loss of on-target activity. Our findings highlight the significance of T-CAST to assess off-target effects of TALEN designer nucleases and to evaluate mitigation strategies, and advocate the use of obligate-heterodimeric TALEN scaffolds for therapeutic genome editing.

## 1 Introduction

Since the advent of CRISPR-Cas technology for genome engineering in 2012 (Gasiunas et al., 2012; Jinek et al., 2012), genome editing has gradually moved towards clinical application (Cornu et al., 2017). Despite being used less frequently in research and development, first in line genome editing tools, such as zinc-finger nucleases (ZFNs) and transcription activator-like effector nucleases (TALEN), have been applied in numerous clinical trials, to treat *inter alia* infection with human immunodeficiency virus (HIV) (Tebas et al., 2014) hematologic malignancies (Qasim et al., 2017; Benjamin et al., 2022), mucopolysaccharidosis and hemophilia (Harmatz et al., 2022), as well as sickle cell disease (NCT03653247). In contrast to RNA-guided nucleases, CRISPR-Cas9 and CRISPR-Cas12a/Cpf1, ZFNs and TALENs act as dimers and are all-protein. They consist of an engineered DNA-binding domain (DBDs) fused to the C-terminal nuclease portion derived from the FokI endonuclease (Porteus and Carroll, 2005; Cathomen and Joung, 2008; Urnov et al., 2010; Gaj et al., 2013). The DBD of a TALEN arm is typically composed of 15–18 TALE repeats, each consisting of 34 amino acids (Christian et al., 2010; Miller et al., 2011; Mussolino et al., 2011). The two amino acids in positions 12 and 13, the so-called repeat-variable di-residues (RVDs), code for the binding specificity of each TALE repeat to a respective DNA base in a simple 1:1 code (Boch et al., 2009; Moscou and Bogdanove, 2009). However, although each RVD typically favors one particular base, there is some promiscuity in binding the DNA. This means that usually more than one base can be recognized by a given RVD (Streubel et al., 2012; Guilinger et al., 2014; Juillerat et al., 2014). To induce a DNA double strand break (DSB), a TALEN pair is designed such that either DBD targets an opposing DNA strand of the target site in a tail-to-tail configuration, separated by a spacer of 10–25 bps (Mussolino et al., 2011; Christian et al., 2012). Upon concomitant binding of both TALEN arms, the two FokI domains dimerize and introduce a staggered-end DNA double strand break (Christian et al., 2010).

In various protocols to manufacture cells for clinical applications, TALENs have proven themselves as a highly efficient gene editing tool, reaching editing efficiencies of >80% in multiple primary human cells (Gautron et al., 2017; Alzubi et al., 2021; Romito et al., 2021; Yang et al., 2022). Also, much effort has been invested to improve the specificity of TALENs for clinical application. This includes the optimization of the length and composition of the linker that connects the TALE to the FokI domain (Mussolino et al., 2011; Christian et al., 2012; Guilinger et al., 2014), the expansion of the RVD repertoire (Juillerat et al., 2015; Miller et al., 2015), as well as preventing homodimerization by creating obligate heterodimeric FokI domains (Miller et al., 2007; Szczepek et al., 2007; Söllü et al., 2010; Doyon et al., 2011; Cade et al., 2012; Nakajima and Yaoita, 2013; Schwarze et al., 2021).

In recent years, genotoxicity, the umbrella term for all unwanted and potentially harmful gene editing events, has become a focal point in the gene editing field aimed towards clinical applications. Several methods have been developed to predict and detect off-target editing, which can be subdivided in three main categories: i) *in silico* prediction of off-target sites based on homology to the target site, ii) methods to determine off-target cleavage *in vitro* and iii) assays to detect off-target effects in cellula, reviewed in (Kim et al., 2019; Wienert and Cromer, 2022).

In silico prediction algorithms for TALENs, like PROGNOS (Fine et al., 2014), compare a given target sequence to the whole genome and return sequences with high similarity, which are therefore likely to be inadvertently targeted. They have the advantage of being easy to use but can suffer from a lack of sensitivity. *In vitro* based methods do not exist for TALENs because a sufficient amount of purified TALEN protein needed for such assays cannot be produced thus far. Cell-based methods and *in situ* assays generally return a low false positive rate, and have been employed successfully to detect TALEN off-target activity (Frock et al., 2015; Romito et al., 2021; Turchiano et al., 2021; Liu et al., 2022). Two of the cell-based assays detect chromosomal rearrangements between the on-target site and an off-target site (Frock et al., 2015; Turchiano et al., 2021) and can use this information to nominate the putative off-target site. CAST-Seq in particular can further classify the structural variations into off-target mediated translocations (OMTs), homology-mediated translocations (HMTs), and large deletions/inversions at the on-target site (Turchiano et al., 2021).

The first CAST-Seq pipeline for the analysis of designer nuclease was based on the analysis of off-target effects of several CRISPR-Cas nucleases and a single TALEN. Revisiting the pipeline with a different TALEN pair revealed some flaws for its use for TALENs. Because the original CAST-Seq pipeline was optimized for evaluation of monomeric CRISPR-Cas nucleases, its use with dimeric TALENs can lead to false classification of translocation events and wrongful annotation of the off-target sites. This prompted us to implement T-CAST as an extension to CAST-Seq, specifically dedicated to and optimized for the analysis of TALEN-mediated off-target events. We improved off-target annotation by implementing a new substitution matrix combined with coverage plot analyses, and validated the T-CAST pipeline with previously published TALEN pairs targeting the clinically relevant loci *CCR5* and *TRAC* (Mussolino et al., 2014; Alzubi et al., 2021). In this context we verified that obligate-heterodimeric TALENs outperformed the wild type scaffold in terms of specificity without impact on on-target activity. Taken together, T-CAST is a novel tool for the unbiased evaluation of on- and off-target effects induced by TALENs, with the possibility to extend its use to other dimeric nucleases.

## 2 Materials and methods

### 2.1 TALEN design and production

All wild type TALEN pairs used were previously described. *TRAC*-targeting TALE nucleases were used in a proof-of-concept study to show feasibility of large scale production of off-the-shelf CAR T cells (Alzubi et al., 2021). TALE nucleases targeting *CCR5* have been investigated in a comparative study benchmarking them against ZFNs (Mussolino et al., 2014). OH-TALEN encoding plasmids were produced in two steps by conventional cloning. First, the wild type FokI domains were excised using restriction enzymes PmeI and BamHI (NEB). Second, FokI domains encoding for OH-substitutions, ordered as gBlocks from IDT, were inserted using the NEBuilder^®^ HiFi DNA Assembly Master Mix following manufacturer instructions. All TALEN-encoding mRNAs were produced by *in vitro* transcription using the HiScribe™ T7 ARCA mRNA Kit with tailing (NEB) following manufacturer instructions. Target sequences of TALENs used in this study are shown in Supplementary Table S1.

### 2.2 PBMC isolation

PBMCs were isolated from leukocyte reduction system (LRS) chambers obtained from the Blood Donation Center (University of Freiburg, Medical Center) by density gradient centrifugation using Ficoll. Appropriate aliquots were resuspended in CryoStor™ CS10 (StemCell Technologies) for long-term storage in liquid nitrogen.

### 2.3 T cell activation and culture

PBMCs/T cells were cultured at 37°C with 5% CO_2_. Upon thawing, PBMCs were washed with PBS (300xg, 5min) and resuspended at a density of 2 × 10^6^ cells/ml in X-VIVO™ 15 (Lonza) medium supplemented with 200U/ml rhIL-2 (Immunotools) and seeded into 24-well plates (1 ml/well). Adherent cells were allowed to attach for 4 h. After 4 h, non-adherent cells were collected, counted, adjusted to a cell density of 1 × 10^6^ cells/ml with X-VIVO™ 15 (Lonza) medium supplemented with 200U/ml rhIL-2 (Immunotools) and re-seeded into 24-well plates (1ml/well). To each well, 5 µl of ImmunoCult™ Human CD3/CD28/CD2 T Cell Activator (StemCell Technologies) was added and the T cells activated for 72–96 h prior to gene editing.

### 2.4 Gene editing of T cells

Prior to gene editing, activation of T cells was assessed by staining for CD25. Downstream experiments were performed only when T cells were highly activated (>85% CD25-expression). For small scale transfer of TALEN mRNAs, 1 × 10^6^ cells were harvested by centrifugation (300xg, 5 min) and the supernatant removed. Cells were resuspended in 50 µl CliniMACS ^®^ Electroporation Buffer (Miltenyi Biotec). Just before electroporation, 7.5 µg of each left and right (or left/left, right/right) TALEN pairs were mixed with the resuspended T cells and electroporated using a CliniMACS ^®^ Prodigy with Electroporator unit (Miltenyi Biotec) with the previously described Setting 3 (Alzubi et al., 2021). Post electroporation, cells were recovered in 400 µl of pre-warmed X-VIVO™ 15 (Lonza) medium supplemented with 200 U/ml rhIL-2 (Immunotools) and seeded into two wells of U-shaped 96-well plates. Half of the cells were subjected to a transient low temperature shift to 32°C for 24 h before being shifted to 37°C. The other half was directly cultured at 37°C. Approximately half of the media was changed every 2 days and the cells split every 3–4 days.

### 2.5 Antibodies and surface staining for flow cytometry

All flow cytometry measurements were carried out using a BD Accuri C6 device. Cells were stained for 45–60 min at 4°C–8°C. T cell activation was assessed by staining with anti-human CD3-APC (Miltenyi Biotec, clone BW264/56) and anti-human CD25-PE (Miltenyi Biotec, clone 4E3). TRAC knock-out was assessed 6–7 days after gene editing by staining with anti-human TCRα/β-PE (Miltenyi Biotec, clone BW242/412) and anti-human CD3-APC (Miltenyi Biotec, clone BW264/56).

### 2.6 Intracellular staining of TALEN

1 × 10^6^ cells were electroporated with 7.5 μg of left and right TALEN mRNAs. After 4 h of incubation at 37°C, 1 × 10^5^ cells were harvested (300xg, 5min) and washed with 500 μL FACS buffer (PBS supplemented with 5% FCS). Cells were subsequently treated with 100 μL BD Cytofix/Cytoperm™ (BD Biosciences). After 30 min incubation on ice, the cells were washed twice with 500 μL BD Perm/Wash Buffer (BD Biosciences). Permeabilized/fixed cells were stained with 50 μL rabbit anti-RVD antibody (Yang et al., 2022) (1:250) *via* incubation for 30 min on ice. After another wash step with 500 μL BD Perm/Wash Buffer, 100 μL of 1:500 diluted secondary goat anti-rabbit antibody (Life Technologies, clone A-11034) was added. Following an incubation for 30 min on ice, the cells were washed once with BD Perm/Wash Buffer and finally FACS buffer. Afterwards, the cells were analyzed using a BD Accuri C6 device.

### 2.7 T-CAST library preparation

Library preparations were basically performed as previously described (Turchiano et al., 2021) using samples that showed particularly high editing as determined by T7E1. In contrast to the original protocol, agarose gel-extraction using the QIAquick Gel extraction kit (QIAGEN) was performed on PCR fragments with a size of 200–500 bp originating from PCRII. This step was included to remove non-informative, short PCR fragments (<200 bp) prior to the barcoding step. NGS libraries were sequenced by Genewiz (part of Azenta Life Sciences) on Illumina HiSeq or NovaSeq with 2 × 150 bp read lengths. Only sites detected as significant hits in two technical replicates are depicted throughout this study. CAST-Seq and T-CAST analysis results for all TALENs targeting *CCR5* and *TRAC* are provided in Supplementary Tables S4–S10. Oligonucleotides used for T-CAST are listed in Supplementary Tables S11.

### 2.8 T-CAST pipeline

Every single replicate, treated and untreated control, is processed independently from the alignment up to the cluster definition, as described in (39). Then, an overlap analysis is performed to unify the clusters from several replicates. Clusters overlapping or separated by less than 1,500 bp are merged and considered as a single translocation event [see (Turchiano et al., 2021) for details]. Based on the number of replicates, the user can define the minimum number of replicates where the site was found, and the minimum number of samples in which the site was significantly different from untreated control (i.e., the number of reads was significantly higher in treated vs. untreated based on Fisher’s exact test).

*Barcode hopping:* We introduced an additional filter to eliminate artifacts generated by barcode hopping events. Barcode hopping are identified by their low reads:hits ratio in comparison to real translocation events by the formula: log10 (reads:hits) distribution (<Q1—2.5*IQ).

*Coverage*: For the remaining sites, the read coverage is calculated in order to identify highly covered regions. Sites are divided into 100 bins of equal size. For each site, the coordinates of bin with the highest coverage across all replicates is used for downstream analysis instead of the whole site coordinates. This new feature restricts the alignment against the target sequence to a smaller, and highly covered region. This makes the alignment more specific and less prone to identification of false-positive OMTs/HMTs.

*Alignment*: A new TALEN-specific substitution matrix was implemented (Supplementary Tables S12) inspired by (18), and analysis restricted to four TALEN combinations: LF.LR, LF.RR, RF.RR, and RF.LR (L/RX, left/right; XF/R, forward/reverse). In order to determine the best combination, i.e., the one that is most likely cleaving an off-target site, different spacer lengths from 8 to 28 bp, are tested for each combination. Artificial sequences, representing binding sites of two TALEN arms separated by a spacer “N_k_” of 8–28 nucleotides (k belong to 8:28) are tested. N can match any bases without cost, therefore the length of the spacer does not influence the alignment score by itself. An example sequence is shown in Supplementary Figure S2B. Alignment score is calculated using the pairwise Alignment function from Biostrings R package with a “local-global” alignment type. The different TALEN combinations and spacer lengths are first selected based on two criteria: a) The first (5′) aligned base is a T, b) the last (3′) aligned base is an A. Then we ordered them based on the alignment score and define the highest score as the most probable TALEN combination and spacer length for a given target site. The same approach was performed on randomly selected regions over the entire genome to determine the overall distribution of the alignment score on random sequences. *p* values of a given combination and spacer length are assessed based on the empirical cumulative distribution function. Sites with *p* values below 0.05 are considered as OMT. HMTs and NBSs were classified in the same way as described in (39).

### 2.9 Amplicon NGS

TALEN target sites as well as putative off-target sites were amplified from 100 ng genomic DNA by standard PCR using Q5^®^ Hot Start High-Fidelity DNA Polymerase (NEB). PCR fragments were purified using either the QIAquick PCR Purification Kit or the QIAquick Gel Extraction Kit (both QIAGEN). Purified PCR products were pooled per sample and NGS libraries constructed using the NEBNext^®^ Ultra™ II DNA Library Prep Kit for Illumina^®^ (NEB). The resulting NGS libraries were quantified by ddPCR using the ddPCR™ Library Quantification Kit for Illumina TruSeq (Bio-Rad) and sequenced on Illumina HiSeq or NovaSeq platforms with 2 × 150 bp read length by NGS service provider Genewiz (part of Azenta Life Sciences). Reads were analyzed using the CRISPResso2 package (Clement et al., 2019) and the *p* values obtained from CRISPResso Compare. All primers used for Amplicon-NGS are listed in Supplementary Tables S11.

### 2.10 On-target activity assessed by T7E1 assay

TALEN target sites were amplified from 100 ng of genomic DNA extracted using the NucleoSpin Tissue Mini Kit for DNA from cells and tissue (Macherey-Nagel) by standard PCR using Q5^®^ Hot Start High-Fidelity DNA Polymerase (NEB). Fragments were purified using the QIAquick PCR Purification Kit (QIAGEN) and subsequently denatured by incubation at 95°C for 5 min. Denatured DNA fragments were allowed to re-anneal through slow cooling of the samples to room temperature. Heteroduplex cleavage as surrogate readout for Indel formation/gene editing was visualized through enzymatic restriction of 100 ng of re-annealed sample with 7.5U of T7 endonuclease I (NEB) for 30 min at 37°C. T7E1 cleavage efficiency was determined by agarose gel electrophoresis and adjacent analysis of the gel images using ImageJ 1.47v.

### 2.11 In silico prediction

In silico prediction of TALEN off-target sites was performed using the PROGNOS web tool (Cradick et al., 2014). For prediction, the individual RVDs were entered and up to six mismatches per TALEN half site allowed. Further, a distance of 10–25 bp between TALEN binding sites was allowed as well as formation of both hetero- and homodimers enabled. PROGNOS results are listed in Supplementary Tables S2 (*CCR5*) and S3 (*TRAC*).

## 3 Results

### 3.1 The need for a specialized CAST-Seq pipeline for TALENs

**FIGURE 1:**
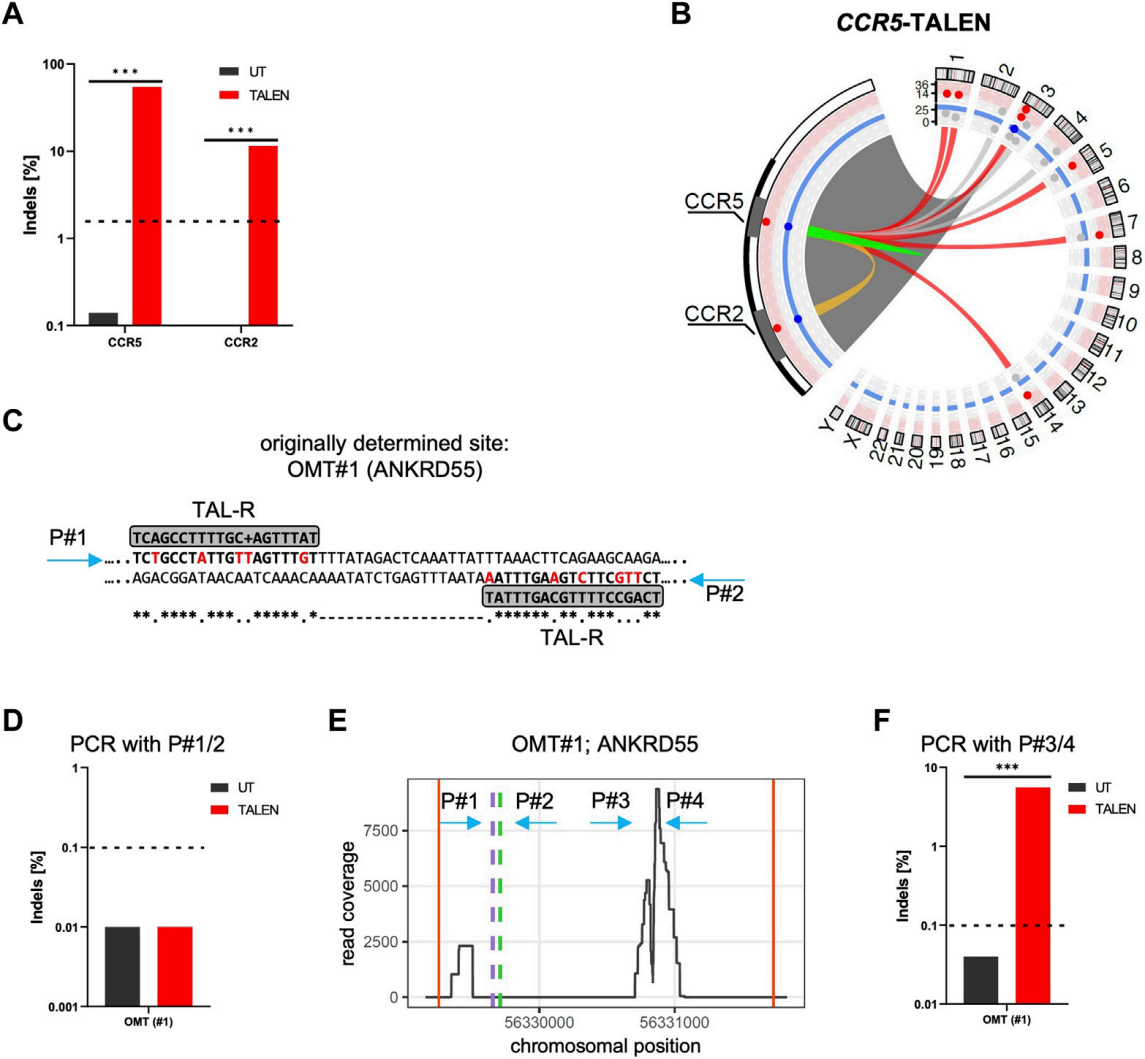
CAST-Seq analysis for *CCR5*-targeting TALEN. **(A)** NGS-based genotyping of *CCR5* and *CCR2*. Indicated is the fraction of alleles bearing Indels at the *CCR5* on-target site and the off-target site in *CCR2* in untreated (UT) T cells, and T cells edited with TALEN. **(B)** Structural variations. Circos plot illustrates CAST-Seq results with enlargement of the chromosome three region encompassing *CCR5* and *CCR2* loci. Lines represent chromosomal rearrangements with the *CCR5* target site: OMTs with >20 hits in red, NBSs with >40 hits in grey, ambiguous classification (OMT/HMT) in yellow. Red and blue layers represent the alignment and homology scores, respectively. **(C)** Alignment. Illustrated is the CAST-Seq alignment for *ANKRD55* (OMT#1) to two right TALEN arms. Mismatched bases are highlighted in red. Positions of primers P#1/P#2 for NGS validation are indicated. **(D)** Genotyping of *ANKRD55*. NGS was performed with primers P#1/P#2. **(E)** Coverage plot of *ANKRD55*. Plot shows chromosomal position vs. number of reads, the putative TALEN binding sites (green and purple dashed lines), as well as the positions of primers P#1/P#2 and P#3/P#4. Red vertical lines show boundary of region. **(F)** Genotyping of OMT#1. NGS was performed with primers P#3/P#4. *** specifies *p*-value<0.001 (Fisher’s exact test, Bonferroni corrected *p*-values).

CAST-Seq is a highly sensitive assay to nominate off-target (OT) sites by identifying gross chromosomal aberrations, such as large deletions and inversions at the on-target sites of designer nucleases. It was applied successfully to samples treated with various CRISPR-Cas9 nucleases (Turchiano et al., 2021; AlJanahi et al., 2022) but, to date, only to a single TALEN pair targeting the *HBB* locus (Turchiano et al., 2021). The fact that the bioinformatic nomination of an off-target site is fundamentally different for a dimeric TALEN pair as compared to a monomeric CRISPR-Cas nuclease, prompted us to revisit OT-activity of previously published TALEN targeting the *CCR5* gene (Mussolino et al., 2014). To this end, TALEN-encoding mRNAs were produced and transferred into primary human T cells expanded from PBMCs. Amplicon NGS revealed that the *CCR5*-targeting TALEN caused small insertions and deletions (Indels) in up to 55% of alleles at the on-target site (Figure 1A). In addition, Indel formation at a frequency of 11.5% was observed at the previously described off-target site in *CCR2* (Figure 1A) (Mussolino et al., 2014). Indeed, alignment of the TALEN target site in *CCR5* with the off-target site in *CCR2* situated 15 kb upstream, revealed a single mismatched binding by the right TALEN arm, while the left TALEN can bind to *CCR2* without mismatches with an optimal spacer of 14 bp with reference to the right arm (Supplementary Figure S1A).

CAST-Seq analysis performed on these samples nominated in addition to *CCR2* six high-scoring off-target mediated translocations (OMTs) harboring >20 CAST-seq hits. In addition to those seven off-target sites, three sites on chromosomes 4 (347 hits), chromosome 3 (66 hits), and chromosome 2 (41 hits) were identified (Figure 1B; Table 1). The current algorithm could neither classify them as OMTs nor as homology-mediated translocations (HMTs) but instead categorized them as natural break sites (NBS). Of note, according to the number of CAST-seq hits, translocations between the *CCR5* on-target site and NBS#1 (347 hits) seems to be more frequent than translocations between *CCR5* and OMT#1 (211 hits) (Table 1). We subsequently probed the predicted TALEN off-target site in OMT#1 for the formation of Indels by targeted amplicon NGS. To this end, we designed primers flanking the site at which two right TALEN arms was proposed to bind (4 or six mismatches, respectively) with an 18 bp spacer (Figure 1C), but did not find signs of TALEN-associated off-target activity (Figure 1D).

**TABLE 1:** CAST-Seq results for *CCR5*-targeting TALEN. Listed are OMTs with >20 hits and NBSs with >40 hits. TALEN combinations: LF, left TALEN arm in forward orientation; LR, left TALEN arm in reverse orientation; RF, right TALEN arm in forward orientation; RR, right TALEN in reverse orientation.

Group	Chr.	Start	End	Reads	Hits	Combination	Closest gene	Annotation
ON	3	46346250	46381495	3124912	12663	LF.RR	CCR5	Promoter (<=1 kb)
OMT #1	5	56329257	56332157	29865	211	RF.RR	ANKRD55	Distal Intergenic
OMT #2	14	94421799	94424738	18761	152	RF.LR	SERPINA11	Distal Intergenic
OMT #3	1	169468106	169468960	7584	67	LR.RR	SLC19A2	Exon
OMT #4	1	82060557	82062621	9351	58	RF.LR	LOC101927434	Distal Intergenic
OMT #5	3	45842530	45845049	8582	41	RR.RR	LZTFL1	Intron
OMT #6	7	131476112	131477853	6576	33	LF.LR	MKLN1	Intron
NBS #1	4	153484591	153487995	46482	347	-	TMEM131L	Intron
NBS #2	3	110813808	110814806	10695	66	-	NECTIN3	Distal Intergenic
NBS #3	2	207462628	207463233	8381	41	-	MYOSLID-AS1	Intron

In order to better understand which chromosomal regions had actually translocated from chromosome 5 (OMT#1) to the on-target site, as well as how the deletion landscape at the on-target site looks, we produced plots showing the read coverage at these chromosomal regions (Figure 1E; Supplementary Figure S1B). The read coverage at the on-target site (Supplementary Figure S1B) revealed large deletions of several kilobases. It furthermore showed two distinct peaks, one at the *CCR5* on-target site and a second peak at the known off-target site in *CCR2*, 15 kb upstream (Supplementary Figure S1B). This observation showcased that read coverage is a strong indicator for nominating an off-target site. In contrast, the predicted TALEN binding site in OMT#1 (Figure 1E green and purple dotted lines) were distant from the area of high coverage, as were the primers initially used to assess Indel formation at this site (Figure 1E arrows P#1 and P#2) We therefore designed primers P#3/P#4 flanking the highly covered region and repeated amplicon NGS, uncovering Indels in 6% of alleles (Figure 1F).

Coverage plots for OMT#2-#6 revealed that in only two cases (OMT#3 and OMT#6) the predicted TALEN off-target sites (green and purple dotted lines) were close to the region with the highest read coverage (Supplementary Figure S1C).

### 3.2 T-CAST nominates TALEN off-target sites with high fidelity

Incited by these results, we set out to improve the accuracy and fidelity of CAST-Seq through development of an optimized pipeline for TALENs called T-CAST. Taking advantage of the strong predictive value of read coverage at off-target sites, T-CAST splits up in a first step each translocated region into 100 bins of equal size. Next, the single bin, with the highest relative read coverage obtained from two (or more) CAST-Seq replicates is identified. A restricted region of ±100 bp from the highest bin was chosen, and the TALEN pair was aligned to this shorter region, using a 5′-T constraint, which is a prerequisite for TALEN-DNA engagement (Cornu et al., 2017). Moreover, to improve the predictive power of identifying the correct off-target site, we implemented a TALEN-specific substitution matrix, which accommodates for the fact that RVDs can bind to multiple DNA bases with varying affinity (Supplementary Table S12).

**FIGURE 2:**
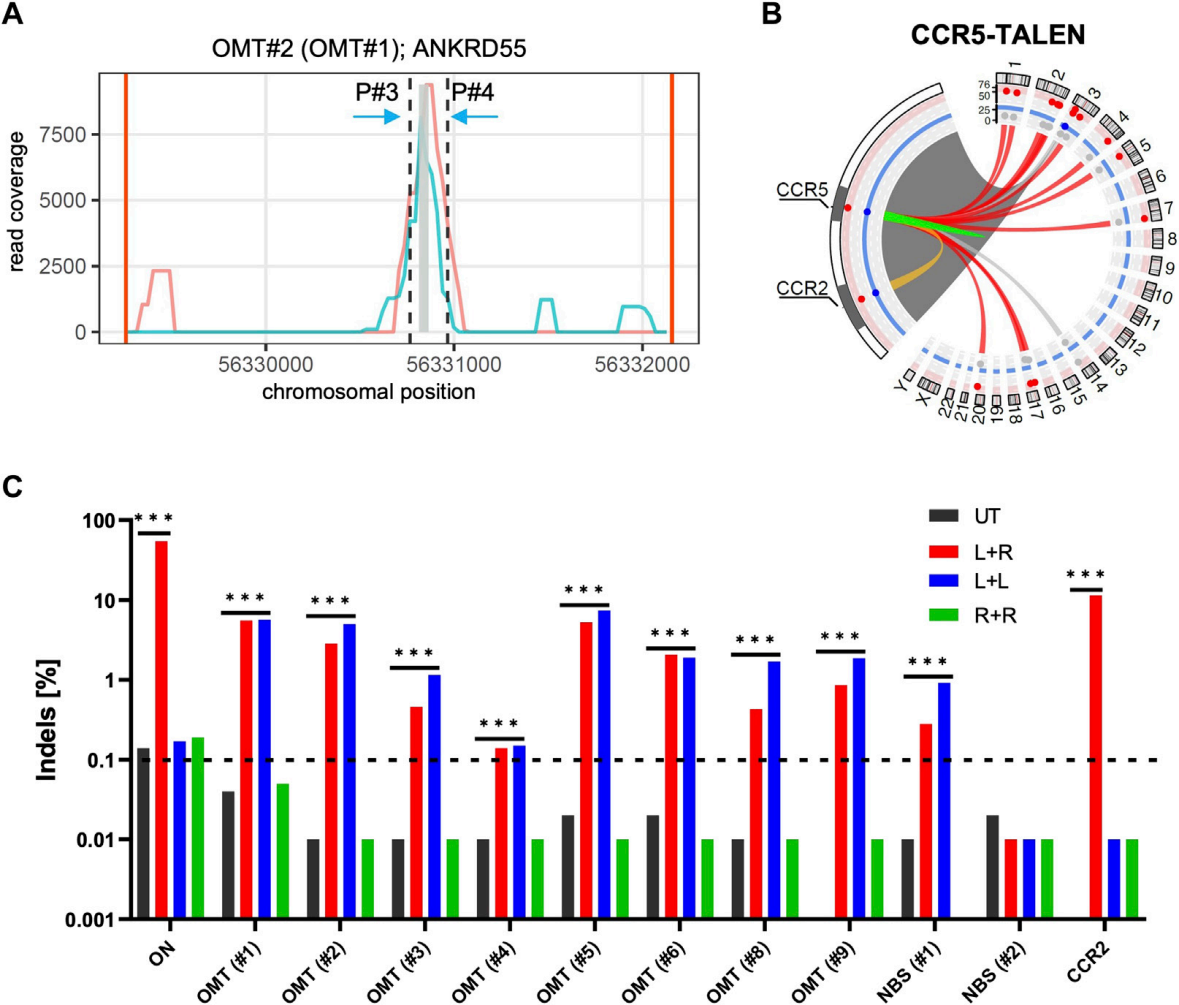
T-CAST analysis for *CCR5*-targeting TALEN **(A)** Coverage plot for *ANKRD55*. Plots showing the coverage of two replicates (turquoise, light red) at the OMT site in *ANKRD55*. Black dashed lines indicate region (±100 bp) flanking the bin with highest coverage (grey). **(B)** Structural variations. Circos plot illustrates T-CAST results with enlargement of the chromosome three region encompassing *CCR5* and *CCR2* loci. Lines represent chromosomal rearrangements with the *CCR5* target site: OMTs with >20 hits in red, NBSs with >40 hits in grey, ambiguous classification (OMT/HMT) in yellow. **(C)** Genotyping. NGS at denoted sites was performed on untreated (UT) T cells, and T cells edited with a combination of left and right TALEN arm (L + R), just left (L + L) or just right (R + R) TALEN arms. *** specifies *p*-value<0.001 (Fisher’s exact test, Bonferroni corrected *p*-values).

As shown in Figure 2A; Supplementary Figure S2A, TALEN binding site nomination is now restricted to the most highly covered regions identified by T-CAST. The implemented changes to the bioinformatics pipeline not only changed the identification of TALEN off-target sites, but also the classification for the observed chromosomal rearrangements. In addition to the known *CCR2* OMT, 12 sites with more than 20 hits were classified as OMTs by T-CAST, on top of two NBS (Figure 2B; Table 2). While the translocation with chromosome 4 (347 hits, Table 1) was now classified as OMT, two sites previously classified as OMTs (OMT#2 and OMT#5) were now re-classified as NBSs (Table 2). Of note, the T-CAST predicted TALEN target site for OMT#2 (ANKRD55, listed as OMT#1 in Table 1) displayed seven mismatches in the first and eight mismatches in the second binding site of the TALEN left arm (Supplementary Figure S2B).

**TABLE 2:** T-CAST results for *CCR5*-targeting TALEN. Listed are OMTs with >20 hits and NBSs with >40 hits. TALEN combinations: LF, left TALEN arm in forward orientation; LR, left TALEN arm in reverse orientation; RF, right TALEN arm in forward orientation; RR, right TALEN in reverse orientation.

Group	Chr.	Start	End	Reads	Hits	Combination	Closest gene	Annotation
ON	3	46372560	46373160	3124912	12663	LF.RR	CCR5	5′ UTR
OMT #1	4	153486313	153486513	46482	347	LF.LR	TMEM131L	Intron
OMT #2	5	56330766	56330966	29865	211	LF.LR	ANKRD55	Distal Intergenic
OMT #3	1	169468608	169468808	7584	67	LF.LR	SLC19A2	Exon
OMT #4	3	110814452	110814652	10695	66	LF.LR	NECTIN3	Distal Intergenic
OMT #5	1	82062263	82062463	9351	58	LF.LR	LOC101927434	Distal Intergenic
OMT #6	2	207462882	207463082	8381	41	LF.LR	MYOSLID-AS1	Intron
OMT #7	17	1834349	1834549	9936	37	RF.LR	RPA1	Intron
OMT #8	2	224106645	224106845	7808	37	LF.LR	SERPINE2	Distal Intergenic
OMT #9	7	131476282	131476482	6576	33	LF.LR	MKLN1	Intron
OMT #10	2	156801241	156801441	4409	33	LF.LR	GPD2	Distal Intergenic
OMT #11	17	37438329	37438529	5278	30	LF.LR	TBC1D3H	Intron
OMT #12	20	63138626	63138826	2568	24	LF.LR	HAR1B	Distal Intergenic
NBS #1	14	94423347	94423547	19256	154	RF.LR	SERPINA11	Distal Intergenic
NBS #2	3	45844659	45844859	8582	41	LF.LR	LZTFL1	Intron

In order to validate the nominated off-target sites, we designed amplicon NGS primers flanking OMTs#1-9 and NBS#1/2 (Table 2). We obtained specific PCR products for all sites except OMT#7. NGS revealed significant Indel formation above background (0.1%) at all sites, except NBS#2 (Figure 2D), with Indel frequencies ranging from 0.12% (OMT#4) to more than 5% (OMT#1 and OMT#5). We challenged the T-CAST prediction for the most likely TALEN conformation to cause off-target activity by transferring mRNA encoding either only the left TALEN arm (L + L) or only the right TALEN arm (R + R) into activated T cells. In line with the T-CAST annotation, formation of Indels at off-target sites was observed in all samples treated with only left TALEN arm but not in cells exposed to the right TALEN arm only (Figure 2C). The exception is off-target activity at *CCR2*, for which both TALEN arms is necessary (L + R). Of note, the coverage plots in Supplementary Figure S1B uncovered all the events in the whole region between the *CCR5* and *CCR2*, thus exposing *CCR2* as off-target combined with large deletions. It is also worth mentioning that with the exception of *CCR2* PROGNOS (Fine et al., 2014) did not predict any of these sites, even when allowing up to six mismatches per TALEN arm (Supplementary Table S2).

### 3.3 Obligate-heterodimeric (OH) TALEN mitigate off-target effects while retaining full on-target activity

**FIGURE 3:**
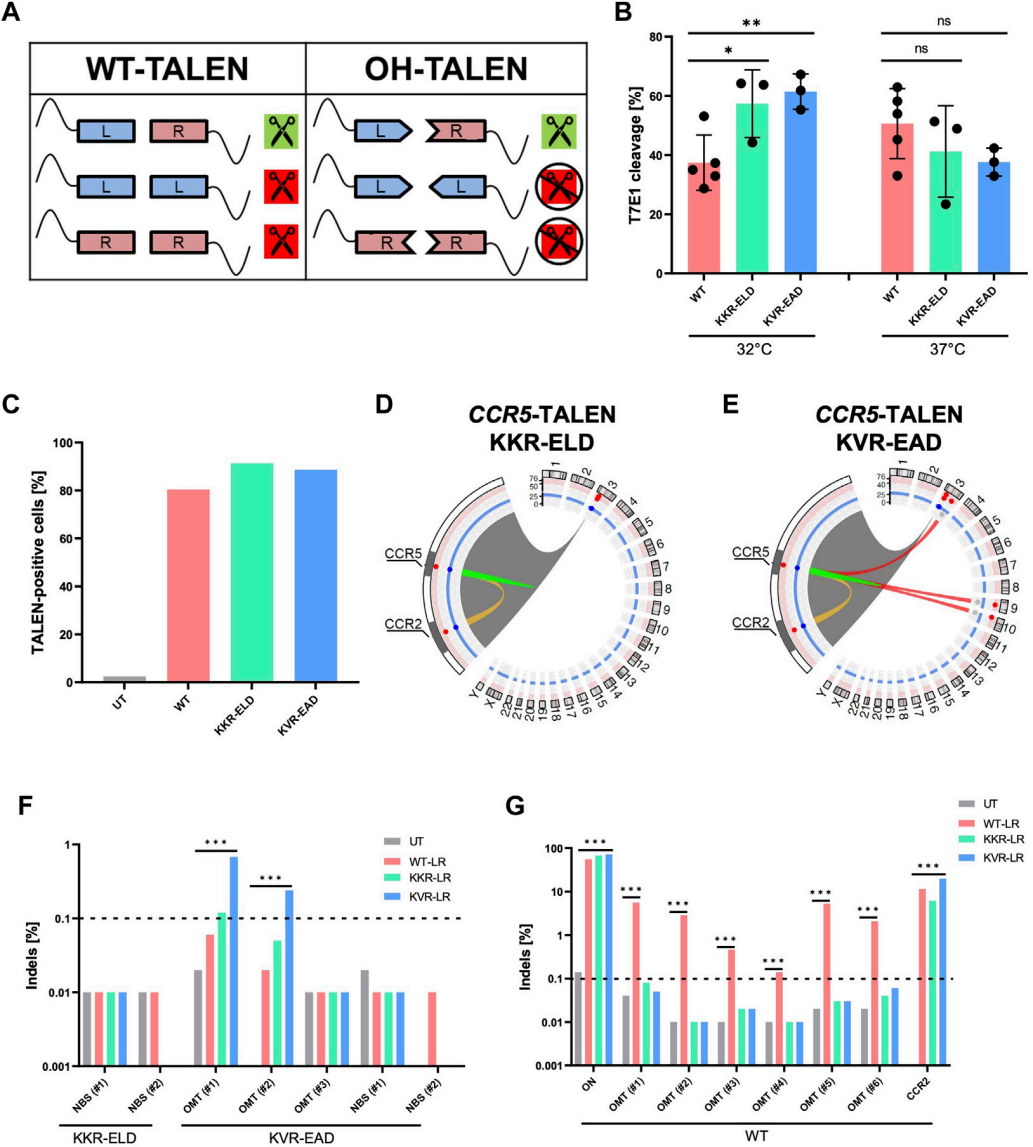
Obligate-heterodimerization mitigates off-target activity of *CCR5*-TALEN **(A)** Schematic of possible TALEN pairing combinations. Combinations are displayed for TALENs with wild-type (WT) and obligate-heterodimeric (OH) FokI domains. **(B)** Genotyping. Results of T7E1 assay of WT-TALEN and OH-TALEN (KKR-ELD and KVR-EAD configuration) are shown. 32°C indicates that T cells were subjected to transient cold-shock after electroporation, while control cells were constantly cultured at 37°C. * and ** specify *p*-values of <0.05 or <0.01 (Student’s t-test, n = 3–5). **(C)** TALEN expression. Percentage of TALEN-expressing T cells upon mRNA transfer as determined by flow cytometry (n = 1). **(D,E)** Structural variations. Circos plots illustrate T-CAST results with enlargement of the chromosome three region encompassing *CCR5* and *CCR2* loci. Lines represent chromosomal rearrangements with the *CCR5* target site: OMTs with >20 hits in red, ambiguous classification (OMT/HMT) in yellow. **(F,G)** Genotyping. NGS at denoted sites was performed on untreated (UT) T cells, and T cells edited with WT-TALENs or OH-TALENs as indicated. The group in which the samples were originally identified is indicated on the bottom. *** specifies *p*-value<0.001 (Fisher’s exact test, Bonferroni corrected *p*-values).

TALEN-mediated DNA cleavage is performed through dimerization of the two FokI domains upon binding in a tail-to-tail orientation of the two subunits. This can be brought about by two different TALEN arms but also through the formation of homodimers as described above (Figure 2C). In order to prevent homodimerization, charged residues within the two FokI dimer interface can be substituted by oppositely charged residues, exerting repellent electrostatic forces and preventing dimer formation of two identical TALEN subunits (Cade et al., 2012; Nakajima and Yaoita, 2013; Schwarze et al., 2021). These obligate-heterodimeric TALEN pairs reduce the number of possible TALEN conformation by 50% (Figure 3A).

Here, we validated two OH-TALEN scaffolds harboring either KKR-ELD or KVR-EAD substitutions in the FokI domain, either linked to the right or to the left TALEN arm. On-target activity of WT and OH-TALENs was assessed on genomic DNA isolated from primary T cells that were cultured constantly at 37°C or subjected to a transient temperature shift to 32°C for 24 h post-electroporation with TALEN-encoding mRNA (Figure 3B). Genotyping by T7E1 assay revealed significantly higher Indel formation for both OH-TALEN scaffolds, averaging 56% and 60% mutated alleles for KKR-ELD and KVR-EAD TALEN, respectively, in contrast to 36% mutated alleles upon transfer of WT TALEN under transient cold-shock (32°C) conditions. Under constant temperature at 37°C, no significant differences were observed between the activities of WT-TALEN and OH-TALEN, with 35%–47% cleavage in T7E1 assay (Figure 3B). Flow cytometric analysis upon intracellular staining 4 h after mRNA transfer revealed similar expression for all TALEN scaffolds (Figure 3C).

Primary T cells that were edited with *CCR5*-targeting OH-TALENs were subsequently subjected to T-CAST analysis. In contrast to WT-TALEN, no high-scoring chromosomal aberrations were observed in samples treated with KKR-ELD TALEN (Figure 3D) and only three structural variations with >20 T-CAST hits were identified in KVR-EAD TALEN treated cells (Figure 3E). Since no chromosomal translocations were detected in KKR-ELD samples, we decided to probe the two top-scoring NBSs (Table 3) for Indel formation. No Indels were detected at these sites in neither WT nor OH-TALEN treated samples (Figure 3F). Likewise, we analyzed all three nominated off-target sites identified in KVR-EAD TALEN treated samples as well as the two highest-scoring NBSs (Table 3) with respect to the presence of Indels by amplicon NGS. Indel formation above the limit of detection were only observed at OMT#1 and OMT#2 in KVR-EAD TALEN treated samples but hardly above background (Figure 3F). In addition, we tested for Indel formation at the six top-scoring OMTs identified previously for WT TALEN (Table 2) as well as the on-target site and the *CCR2* off-target site. In line with the T7E1 results, the fraction of mutated alleles ranged from 56% (WT) to 68% and 72% for KKR-ELD and KVR-EAD, respectively (Figure 3G). Off-target mutagenesis in *CCR2* was highest in KVR-EAD edited samples (20%) and lowest in KKR-ELD treated samples (6%). In line with off-target activity at *CCR2*, we detected a large 15 kb deletion between the *CCR5* and *CCR2* loci in all samples, irrespective of the scaffold (Supplementary Figure S3). In conclusion, both OH scaffolds greatly improved the specificity of the *CCR5*-targeted TALEN by abrogating activity at off-target sites that were bound by a single TALEN arm in a homodimeric tail-to-tail configuration.

**TABLE 3:** T-CAST results for *CCR5*-targeting OH-TALEN. Listed are OMTs with >20 hits and the two top scoring NBSs. TALEN combinations: LF, left TALEN arm in forward orientation; LR, left TALEN arm in reverse orientation; RF, right TALEN arm in forward orientation; RR, right TALEN in reverse orientation.

Scaffold	Group	Chr.	Start	End	Reads	Hits	Combi	Closest gene	Annotation
**KKR-ELD**	ON	3	46372773	46373373	2520999	8224	LF.RR	CCR5	Exon
	NBS #1	3	46324767	46324967	4080	17	LF.RR	CCR2	Distal Intergenic
	NBS #2	3	12744579	12744779	5255	9	RF.LR	TMEM40	Intron
**KVR-EAD**	ON	3	46372892	46373492	3068893	11259	LF.RR	CCR5	Exon
	OMT #1	3	136332505	136332705	9510	45	RF.LR	PCCB	Intron
	OMT #2	10	13181066	13181266	4331	30	RF.LR	MCM10	Intron
	OMT #3	9	68584925	68585125	3821	24	RF.RR	LINC01506	Intron
	NBS #1	3	45844643	45844843	9103	38	LF.LR	LZTFL1	Intron
	NBS #2	16	54414079	54414279	4671	24	RF.RR	LINC02140	Distal Intergenic

### 3.4 T-CAST unravels off-target activity of TRAC-targeting TALEN used in large-scale production of universal CAR T cells

**FIGURE 4:**
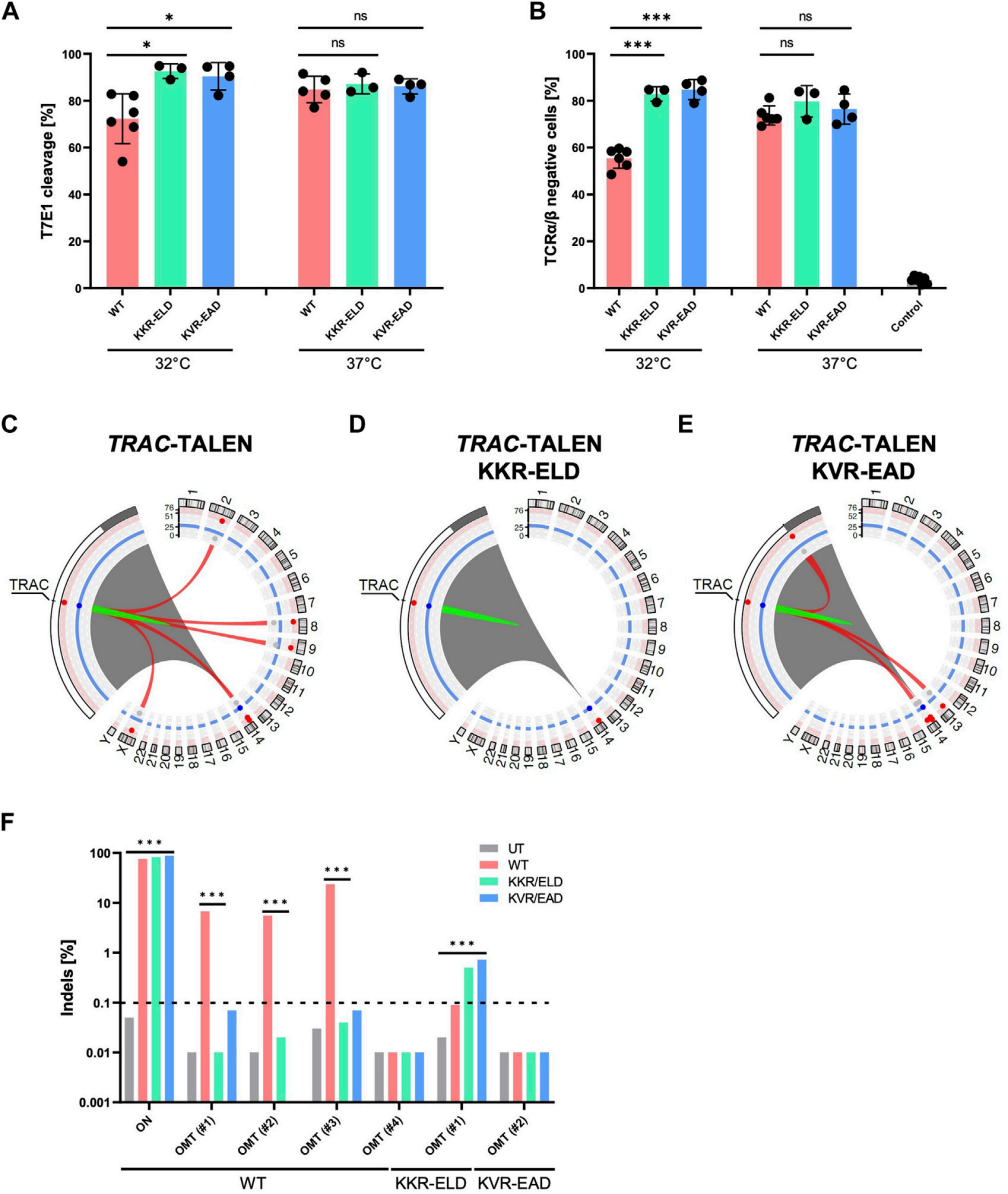
T-CAST analysis for *TRAC*-targeting TALENs **(A)** Genotyping. Results of T7E1 assay of WT-TALEN and OH-TALEN (KKR-ELD and KVR-EAD configuration) are shown. 32°C indicates that T cells were subjected to transient cold-shock after electroporation, while control cells were constantly cultured at 37°C. * specifies *p*-value<0.05 (Student’s t-test, n = 3–6). **(B)** TCR expression. Displayed is the fraction of TCRα/β-negative T cells upon transfer of TALEN-encoding mRNA as determined by flow cytometry. Where indicated (32°C) T cells were subjected to a transient cold-shock. *** specifies *p*-value<0.001 (Student’s t-test; n = 3–7). **(C–E)** Structural variations. Circos plots illustrate T-CAST results with enlargement of the chromosome 14 region encompassing *TRAC*. Red lines represent chromosomal rearrangements (OMTs with >20 hits) with the *TRAC* target site. **(F)** Genotyping. NGS at denoted sites was performed on untreated (UT) T cells, and T cells edited with WT-TALENs or OH-TALENs as indicated. The group in which the samples were originally identified is indicated on the bottom. *** specifies *p*-value<0.001 (Fisher’s exact test, Bonferroni corrected *p*-values).

The constant region of the T cell receptor α chain (*TRAC*) is an interesting target in CAR T cell immunotherapy. Disruption of *TRAC* results in the loss of expression of the entire T cell receptor complex (TCR), a major road block in allogeneic CAR T cell therapy (Lin et al., 2021). We have previously shown that a TALEN targeting *TRAC* can be used in large scale manufacturing of universal CAR T cells (Alzubi et al., 2021). Using T-CAST, we revisited the safety profile of the previously used WT-TALEN in comparison to OH-TALEN scaffolds. Genotyping by T7E1 assays revealed high on-target activity for all three TALEN scaffolds, each one achieving some 90% of T7E1 cleavage (Figure 4A). In line with our findings for *CCR5*-targeting TALENs, OH-TALEN targeting *TRAC* outperformed their WT counterpart when the primary T cells were subjected to a transient 32°C cold-shock post electroporation. These findings are mirrored by the phenotypic analysis of TCRα/β surface expression performed 1 week post-transfer of TALEN-encoding mRNA. Under transient cold-shock conditions, the fraction of TCRα/β-negative cells increased from 55% TCR-negative T cells upon transfer of WT-TALENs to 85% and 83% in T cells edited with KVR-EAD and KKR-ELD OH-TALENs respectively (Figure 4B). T cells cultured at 37°C revealed no significant differences with regard to knockout efficacies between the various scaffolds (Figures 4A, B). A representative flow cytometric analysis (Supplementary Figure S4) shows the two clearly separated populations of TCRα/β positive and TCRα/β negative cells, in agreement with monoallelic expression of the T cell receptor α chain.

Similar to our observations for TALEN targeting *CCR5*, the number of chromosomal rearrangements was highest in samples treated with the WT-TALEN scaffold (Table 4). T-CAST identified a total of five translocations with more than 20 T-CAST hits (Figure 4C). In KKR-ELD TALEN treated samples, no structural variation was identified (Figure 4D), while T cells edited with KVR-EAD TALEN displayed three chromosomal aberrations (Figure 4E). Of note, the highest-scoring rearrangement observed in the KVR-EAD samples, OMT#1 with 79 T-CAST hits, is in close proximity to the on-target site, thus likely representing a large deletion rather than an off-target site. A similar phenomenon was observed in KKR-ELD edited T cells (OMT#2, Table 4).

**TABLE 4:** T-CAST results for *TRAC*-targeting wild type and OH-TALEN. Listed are OMTs with >20 hits or the two top scoring OMTs (KKR-ELD) and NBSs with >40 hits. TALEN combinations: LF, left TALEN arm in forward orientation; LR, left TALEN arm in reverse orientation; RF, right TALEN arm in forward orientation; RR, right TALEN in reverse orientation. Large deletions (Del.) at the *TRAC* on-target site are specified.

Scaffold	Group	Chr.	Start	End	Reads	Hits	Combi	Closest gene	Annotation	Del.
**WT**	ON	14	22547244	22547844	2828806	11658	LF.RR	DAD1	Exon	–
	OMT #1	9	95910827	95911027	55113	193	RF.RR	ERCC6L2	Intron	–
	OMT #2	8	25327743	25327943	35183	189	LF.LR	DOCK5	Intron	–
	OMT #3	2	161944093	161944293	8931	39	LF.LR	SLC4A10	Intron	–
	OMT #4	X	72603585	72603785	3734	38	LF.RR	PHKA1	Intron	–
	OMT #5	14	20970240	20970440	7760	33	RF.RR	RNASE2	Distal Intergenic	–
**KKR-ELD**	ON	14	22547411	22548011	1703820	8651	LF.RR	DAD1	Exon	–
	OMT #1	2	88071594	88071794	3458	17	LF.RR	SMYD1	Intron	–
	OMT #2	14	22574148	22574348	5919	15	LF.LR	DAD1	Intron	Yes
**KVR-EAD**	ON	14	22547381	22547981	1685712	11361	LF.RR	DAD1	Exon	–
	OMT #1	14	22564947	22565147	8375	79	RF.LR	DAD1	3′ UTR	Yes
	OMT #2	14	68540214	68540414	2539	32	RF.LR	RAD51B	Exon	–
	OMT #3	13	22304862	22305062	3585	25	RF.LR	LINC00540	Distal Intergenic	–

Validation of T-CAST results by targeted amplicon NGS confirmed high on-target activity for all TALEN scaffolds, with 76%–88% Indels at *TRAC* (Figure 4F). Assessment of off-targets OMT#1–4 identified for WT-TALEN confirmed off-target activity at OMT#1–3, with 6%–24% Indels at those sites in the WT-TALEN treated samples and absence of off-target mutagenesis in T cells edited with the OH-TALEN scaffolds. No Indels were observed at OMT#4 in any of the edited samples (Figure 4F), while OMT#5 could not be amplified. We furthermore probed OMTs associated with TALEN (OMT#1, 17 CAST-hits) and KVR-EAD TALEN (OMT#2, 32 CAST-hits). Despite the low number of T-CAST hits, a small but significant fraction of reads in both OH-TALEN edited T cell samples revealed off-target activity at this off-target site on chromosome 2 with 0.5%–0.7% of alleles with Indels. No Indels were detected at KVR-EAD OMT#2 (Figure 4F). Of note, similarly to the *CCR5*-targeting TALENs, there is no overlap between the PROGNOS-predicted sites for *TRAC*-targeting TALEN (Supplementary Table S3) and experimentally confirmed off-target sites identified by T-CAST.

In conclusion and in agreement with the results for the *CCR5*-targeting TALENs, both OH scaffolds greatly improved the specificity of the *TRAC*-targeted TALEN by abrogating off-target activity at sites that are cleaved by a homodimeric TALEN.

## 4 Discussion

Gene editing tools have entered the clinical stage almost 10 years ago. Some 50 active interventional clinical trials (ClinicalTrials.gov, accessed on Dec. 22, 2022) employ genome editors to treat devastating diseases through *ex vivo* or *in vivo* applications. While the CRISPR-Cas system is by far the most frequently used platform, other customizable nucleases, such as TALENs or ZFNs are used as well.

With an ever-increasing number of gene editing clinical trials, the demand for highly sensitive assays to detect unwanted side effects is equally on the rise. While the safety of CRISPR-Cas nucleases can be assessed by *in vitro* methods (Tsai et al., 2017), such assays are not transferrable to TALENs and ZFNs because these proteins cannot be produced in sufficient amounts needed for these assays. Moreover, in contrast to CRISPR-Cas, for which several *in silico* off-target prediction tools exist (Cradick et al., 2014; Montague et al., 2014; Park et al., 2015; Stemmer et al., 2015; Haeussler et al., 2016), off-target prediction algorithms are rather scarce for TALENs and they have neither been further developed nor updated in many years (Fine et al., 2014). It is therefore not surprising that the prediction algorithm PROGNOS was not able to forecast most of the off-target sites identified in this study. It is thus highly relevant to have powerful off-target detection methods at hand, which can be applied in cellula. While some assays rely on the concomitant delivery of short DNA fragments to tag DSBs (Tsai et al., 2015), others use chromatin-immunoprecipitation of DNA repair factors to nominate the off-target sites (Wienert et al., 2019). CAST-Seq and other assays (Frock et al., 2015; Turchiano et al., 2021; Liu et al., 2022) identify off-target sites through detection of chromosomal rearrangements triggered by the expression of designer nucleases. These methods are of eminent importance, not only to develop and implement safe therapeutic genome editing strategies but also to monitor the patients during the clinical follow-up phase.

Here, we described the implementation of T-CAST, a CAST-Seq based pipeline that has been optimized to identify off-target effects triggered by TALENs. While CAST-Seq based detection of chromosomal rearrangements is agnostic to the designer nuclease platform, the nomination of the off-target sites is not. The annotation of TALEN off-target sites is particularly challenging because TALE RVDs are–despite showing a strong preference for a particular base–rather promiscuous in their DNA binding behavior (Miller et al., 2011; Streubel et al., 2012; Guilinger et al., 2014; Juillerat et al., 2014). In order to improve the reliability of TALEN off-target site nomination, we took advantage of the fact that the CAST-Seq read coverage at a given chromosomal region is a strong indicator for the presence of an off-target site. The T-CAST pipeline restricts TALEN alignment to this region, and it furthermore accounts for the requirement of 5′-T for efficient DNA binding by a TALEN subunit (Boch et al., 2009; Moscou and Bogdanove, 2009). This current T-CAST pipeline will hence not work properly if TALENs bearing a modified N-terminal domain to recognize all bases at the 5′-end (Lamb et al., 2013) are employed. However, T-CAST can be readily adapted for novel RVD scaffolds and also alternative TALE formats if needed.

T-CAST identified various off-target mediated structural variations triggered by the expression of TALENs designed to target *CCR5* or *TRAC*. Almost all of the nominated off-target sites could be verified by targeted amplicon NGS. In three cases, we did not detect Indels at a predicted OMT. The most likely explanation for this discrepancy is the lower limit of detection (LLOD) of CAST-Seq *versus* targeted amplicon sequencing. While the LLOD of CAST-Seq was determined to be about 0.01%, the LLOD of safely detecting Indels by amplicon NGS is about 10-fold higher. However, we cannot exclude with certainty that the three sites classified as OMTs are false positives. In another instance, we detected Indel formation at a site classified as NBS, suggesting that although T-CAST was able to identify the chromosomal translocation, the classification into an NBS was incorrect. The erroneous classification is founded on the aforementioned fact that RVDs are promiscuous: the T-CAST classifier is unhinged if a TALEN arm accepts seven or more mismatches.

Having set up a reliable bioinformatics pipeline to identify TALEN off-target sites, we used T-CAST to evaluate whether obligate-heterodimeric TALEN scaffolds can mitigate off-target activity. We established that OH-TALENs displayed similar or higher on-target activity as WT TALENs. Interestingly, OH-TALEN outperformed WT-TALEN whenever the cells were subjected to a transient cold-shock post-nucleofection. In contrast, no significant differences were observed when the primary T cells were constantly cultured at 37°C. Since we demonstrated comparable expression levels of the different TALEN scaffolds, we speculate that the diverging behavior is due to an altered affinity between the FokI dimer interfaces of the three TALEN scaffolds tested here.

Regardless, our main focus was to validate reduced off-target activity of OH-TALEN scaffolds. Indeed, the occurrence of chromosomal aberrations in T cells edited with KKR-ELD and KVR-EAD TALENs was strongly reduced. They were detected only at off-target sites that were engaged by a left and a right TALEN subunit, confirming that the OH-TALEN scaffolds effectively prevent off-target events at ‘homodimeric binding sites’. Our observations are consistent with other studies that reported a decrease in OT-activity when using obligate-heterodimeric nucleases (Miller et al., 2007; Szczepek et al., 2007; Söllü et al., 2010; Doyon et al., 2011; Cade et al., 2012; Nakajima and Yaoita, 2013; Schwarze et al., 2021).

In summary, our results show that T-CAST is genome-wide and sensitive method to detect chromosomal rearrangements induced by TALENs, and to nominate TALEN off-target sites with high precision in primary human cells. It can easily be envisioned to adapt T-CAST to additional dimeric genome editing platforms, including ZFNs, double nickase approaches, or other customized endonucleases, for which *in silico* or *in vitro* methods are challenging or not possible. Moreover, as shown for CAST-Seq, T-CAST can be performed on any primary cell type with only 500 ng of genomic DNA required, thus proving its value to monitor chromosomal aberrations during preclinical development, as well as monitoring final product and patients during the follow-up phase.

## Data Availability

The datasets presented in this study can be found in online repositories. The names of the repository/repositories and accession number(s) can be found below: https://www.ncbi.nlm.nih.gov/geo/query/acc.cgi?acc=GSE222763.
