# Supplementary material for: T-CAST: An optimized CAST-Seq pipeline for TALEN confirms superior safety and efficacy of obligate-heterodimeric scaffolds
Source: Front Genome Ed. 2023 Feb 20;5:1130736. doi: 10.3389/fgeed.2023.1130736 (PMC9986454; doi:10.3389/fgeed.2023.1130736)
Supplement: Supplementary file 1 [file DataSheet1.pdf]

Supplementary Material

T-CAST: An optimized CAST-Seq pipeline for TALEN confirms superior safety and efficacy of obligate-heterodimeric scaffolds

Manuel Rhiel†[1,2], Kerstin Geiger †[1,2,3], Geoffroy Andrieux [4,5,6], Julia Rositzka [1,2], Melanie Boerries[4,5,6,7], Toni Cathomen[1,2,6], Tatjana I Cornu* [1,2,6]

[1] Institute for Transfusion Medicine and Gene Therapy, Medical Center-University of Freiburg, 79106 Freiburg, Germany.

[2] Center for Chronic Immunodeficiency (CCI), Medical Center-University of Freiburg, 79106 Freiburg, Germany.

[3] Ph.D. Program, Faculty of Biology, University of Freiburg, 79104 Freiburg, Germany

[4] Institute of Medical Bioinformatics and Systems Medicine, Medical Center-University of Freiburg, Faculty of Medicine, University of Freiburg, Freiburg, Germany.

[5] German Cancer Consortium (DKTK), Partner Site Freiburg, and German Cancer Research Center (DKFZ), Heidelberg, Germany.

[6] Faculty of Medicine, University of Freiburg, 79106 Freiburg, Germany.

[7]Comprehensive Cancer Center Freiburg (CCCF), Medical Center – University of Freiburg, 79106 Freiburg, Germany

Correspondence: Corresponding Author Tatjana.Cornu@uniklinik-freiburg.de

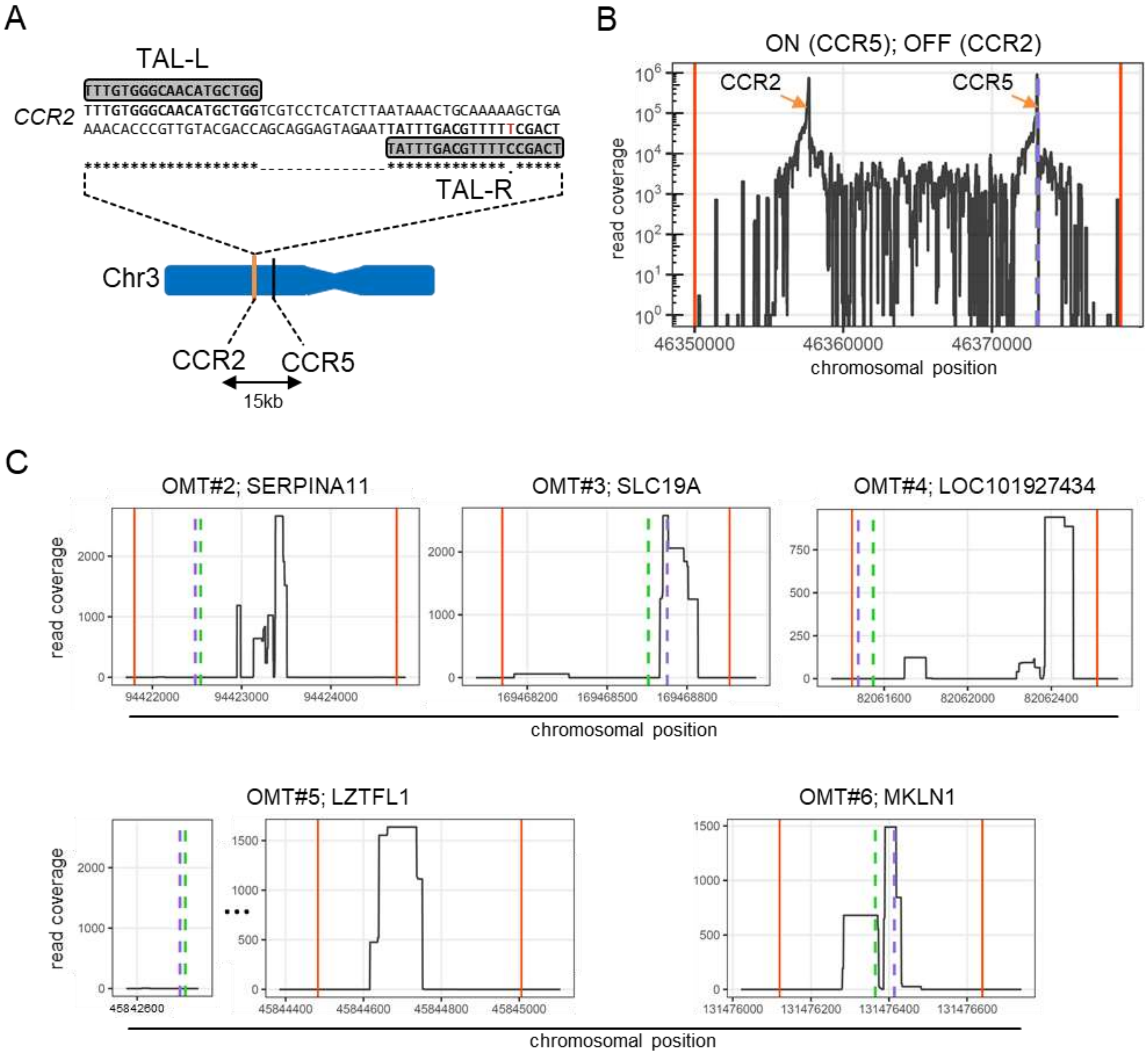

Supplementary Figure 1. CAST-Seq read coverage at on- and off-target sites of CCR5-targeting TALEN

(**A**) Alignment. Illustrated is the CAST-Seq alignment for *CCR2* to both TALEN arms. Mismatched bases are highlighted in red letters, the relative location with respect to the *CCR5* target site is illustrated on the bottom. (**B**) Coverage plot for the *CCR5*-*CCR2* region. Plot shows chromosomal position vs. number of reads. (**C**) Coverage plots for off-target regions. Green and purple dotted lines indicate the putative TALEN binding sites predicted by CAST-Seq.

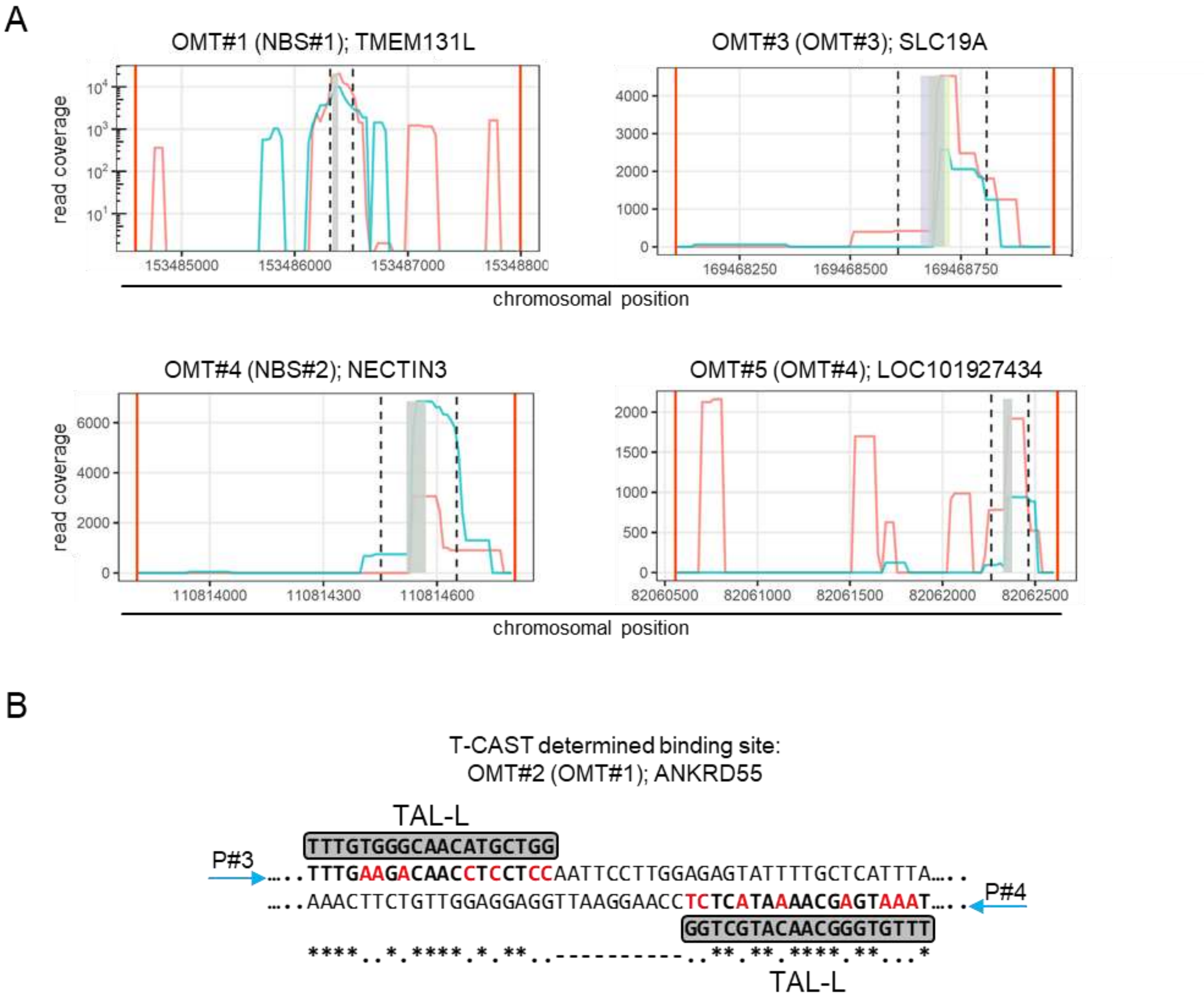

Supplementary Figure 2. Coverage plot-based nomination of off-target sites by T-CAST

(**A**) Coverage plots for off-target regions. Black dotted lines indicate the region (±100 bp) flanking the bin with highest coverage (grey). Plots show coverage of two replicates (turquoise, light red) at indicated OMTs. The former nomination based on the CAST-Seq is given in brackets. Red vertical lines denote boundary of entire region. (**B**) Alignment. Illustrated is T-CAST based alignment for *ANKRD55* (OMT#2) to two left TALEN arms. Mismatched bases are denoted by dots, primer binding sites used for validation (P#3/P#4) are indicated by arrows.

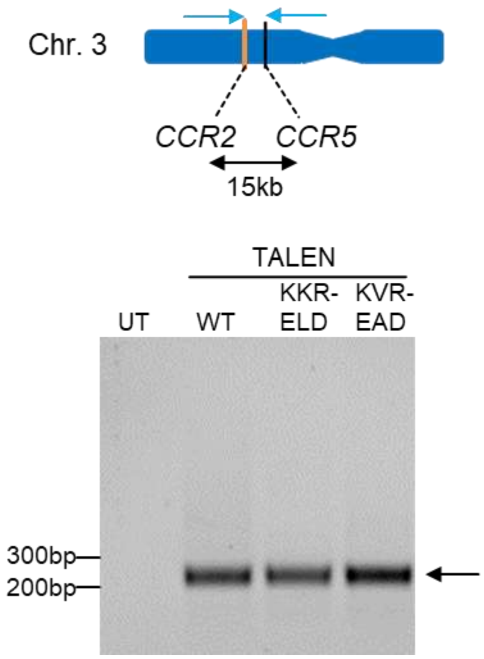

Supplementary Figure 3. Large deletion between *CCR5* and *CCR2*

Schematic specifying the PCR primer positions (blue arrows) used to amplify the *CCR2-CCR5* junction is shown on top. PCR products were analyzed by agarose gel electrophoresis. The positions of the expected PCR amplicon (arrow) and size markers are indicated.

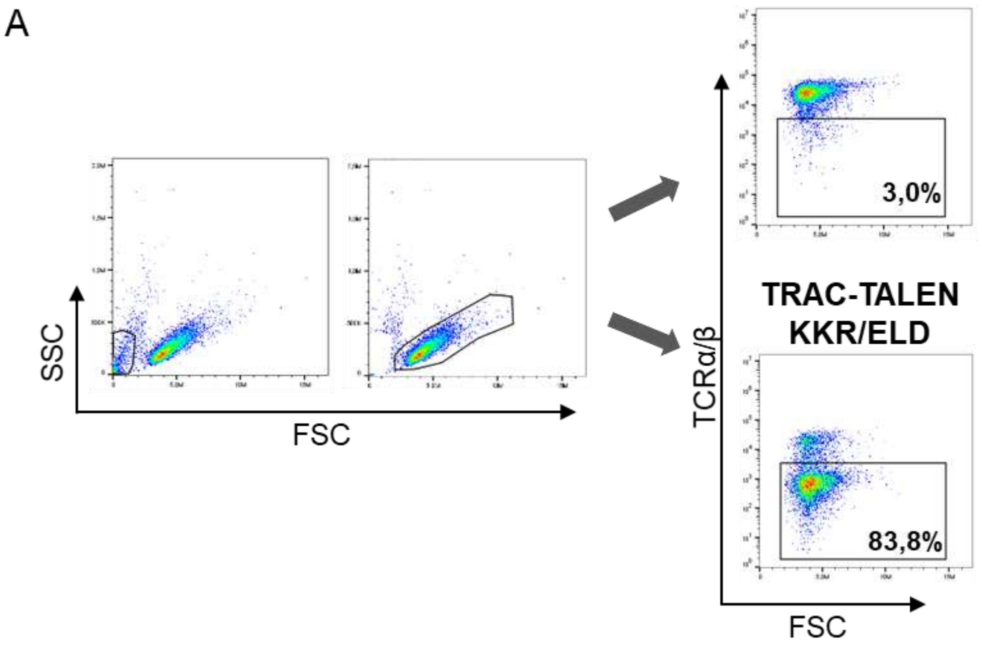

Supplementary Figure 4. TALEN-mediated *TRAC* knockout

(**A**) Gating strategy. Shown is a representative flow cytometry analysis to assess the reduction in TCRα/β surface expression after disruption of *TRAC*. Debris was excluded through gating, and TCRα/β expression was assessed in living cells.

Supplementary Tables

S1: Target sites and RVDs of CCR5 and TRAC TALEN

S2: Prognos analysis of CCR5 TALEN

S3: Prognos analysis of TRAC TALEN

S4: CAST-Seq output analysis of CCR5 WT TALEN

S5: T-CAST output file of CCR5 WT TALEN

S6: T-CAST output file of CCR5 KKR-ELD TALEN

S7: T-CAST output file of CCR5 KVR-EAD TALEN

S8: T-CAST output file of TRAC WT TALEN

S9: T-CAST output file of TRAC KKR-ELD TALEN

S10: T-CAST output file of TRAC KVR-EAD TALEN

S11: Oligonucleotides used in this study

S12: T-CAST substitution matrix
